# Bibliometric mapping for current and potential collaboration detection

**DOI:** 10.5195/jmla.2019.764

**Published:** 2019-10-01

**Authors:** Jordan Wrigley, Virginia Carden, Megan von Isenburg

**Affiliations:** Master’s of Science in Library Science Graduate Student, University of North Carolina–Chapel Hill, and Intern, Library & Archives, Duke University Medical Center, 10 Searle Drive, DUMC 3702, Durham, NC 27710, jeliwrig@gmail.com; Research Impact and EndNote Consultant, Library & Archives, Duke University Medical Center, 10 Searle Drive, DUMC 3702, Durham, NC 27710, virginia.carden@duke.edu; Associate Dean for Library Services & Archives, Library & Archives, Duke University Medical Center, 10 Searle Drive, DUMC 3702, Durham, NC 27710, megan.vonisenburg@duke.edu

## Abstract

This project characterized current research and collaboration patterns in pain research at one institution after researchers working on a grant application approached the library to better understand current institutional research and publishing about that topic. To address this question, library staff developed a collaborative, multi-tool process for bibliometric analysis and network visualization. The primary data source used was a preexisting, curated EndNote library of institutional publications. This EndNote library was searched using keywords relevant to the topic in order to create two sublibraries: one on pain and one specifically on musculoskeletal pain. Article data from each library were exported into InCites to create a benchmarking analysis. In addition, article data were imported into VOSviewer to visualize collaboration networks by author and create concept maps. Researchers were consulted to identify and label resulting clusters in the VOSviewer visualizations. This project successfully generated useful visualizations via bibliometric mapping that characterized current and potential pain research at the institution. The analysis was included in a grant proposal for funding a center for pain research and for catalyzing further collaborative research.

In 2018, researchers working on a grant application approached Duke University Medical Center Library & Archives to better understand current institutional research and publishing about pain among Duke researchers. Because no specific research impact or bibliometrics service existed in the library, librarians worked as an innovative and agile team, pooling knowledge and resources to identify existing data and tools to address these questions. This allowed us to use a combination of preexisting resources with new and novel data visualization tools.

After initial consultations with the researchers, we identified three questions for analysis centered on Duke pain researchers:

1. Who is publishing on pain?
2. Who is collaborating on pain research?
3. What patterns of collaboration between basic, clinical, or translational science researchers exist?

We met to pool skills and resources and determine the best tools to answer these questions. Pooled skills included publication data management, bibliometric and impact analysis, and data visualization. One librarian acted as primary point of contact and maintained iterative dialogue with researchers throughout the project.

We identified the source of publication data for analysis in a preexisting EndNote library of Duke School of Medicine publications that had been developed and was maintained by a Duke medical librarian. This EndNote library was searched using relevant keywords to create two sublibraries: general pain and musculoskeletal pain. Publication data from each were exported into InCites Benchmarking & Analytics to create a benchmarking analysis. InCites is a web-based research evaluation tool, which is available by subscription, for measuring and analyzing institutional productivity via bibliometrics indicators. Publications were ranked in InCites by document and citation count to highlight existing institutional successes in pain research and publishing.

To detect current and potential collaborators within the topic, we extracted full citations for all records from the EndNote library and imported them into the bibliometric mapping and network visualization tool, VOSviewer. VOSviewer (version 1.6.10) is an open source software tool for constructing and visualizing bibliometric networks using publication and other data types [1]. In VOSviewer, the closer data points/nodes are, the more closely related they are [2].

Using VOSviewer, initial term maps and coauthorship networks were generated. Topical areas in pain research were explored through terms text-mined from article titles and abstracts via VOSviewer (Figure 1). Terms (visualized as nodes) are connected by their co-occurrence (visualized as lines). Collaboration was also explored via coauthorship networks among Duke pain researchers. In the visualized coauthorship network (Figure 2), authors are shown as nodes based on data from the curated EndNote library (single asterisk [*] denotes a Duke author; double asterisk [**] denotes a Duke last author) and coauthorship as lines. These visuals also include non-Duke collaborators who have published with Duke authors in the dataset.

**Figure 1:**
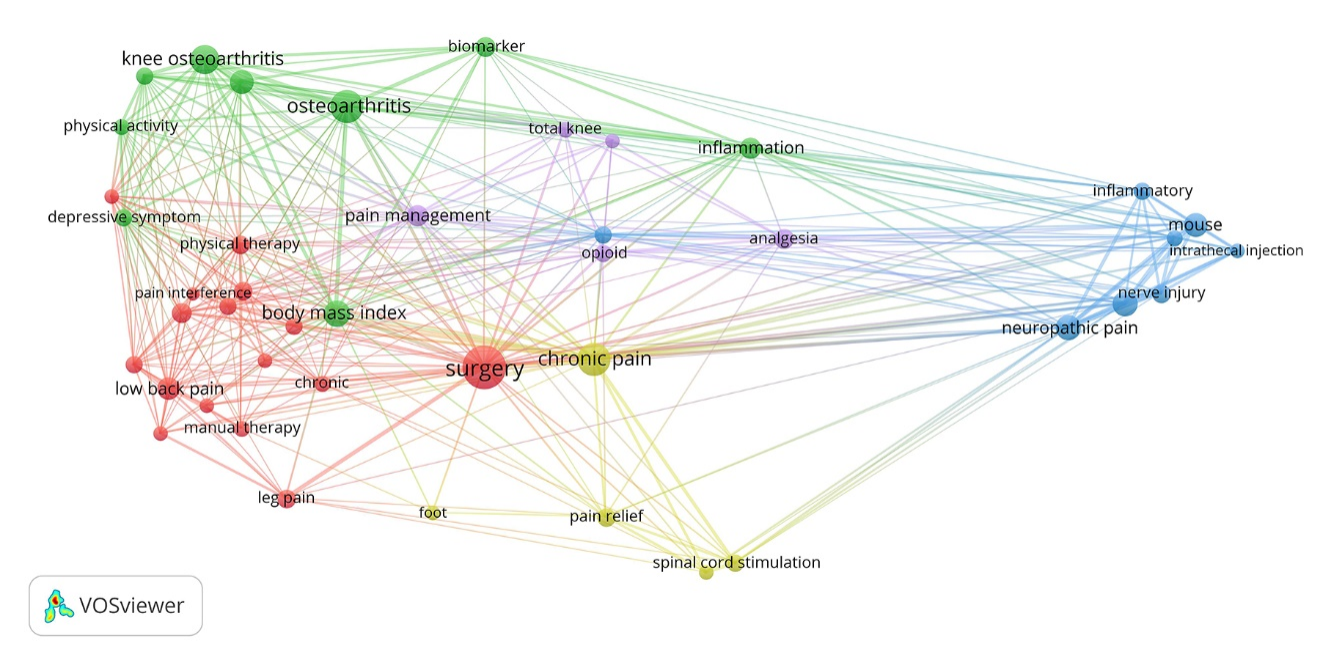
Topical areas in pain research

**Figure 2:**
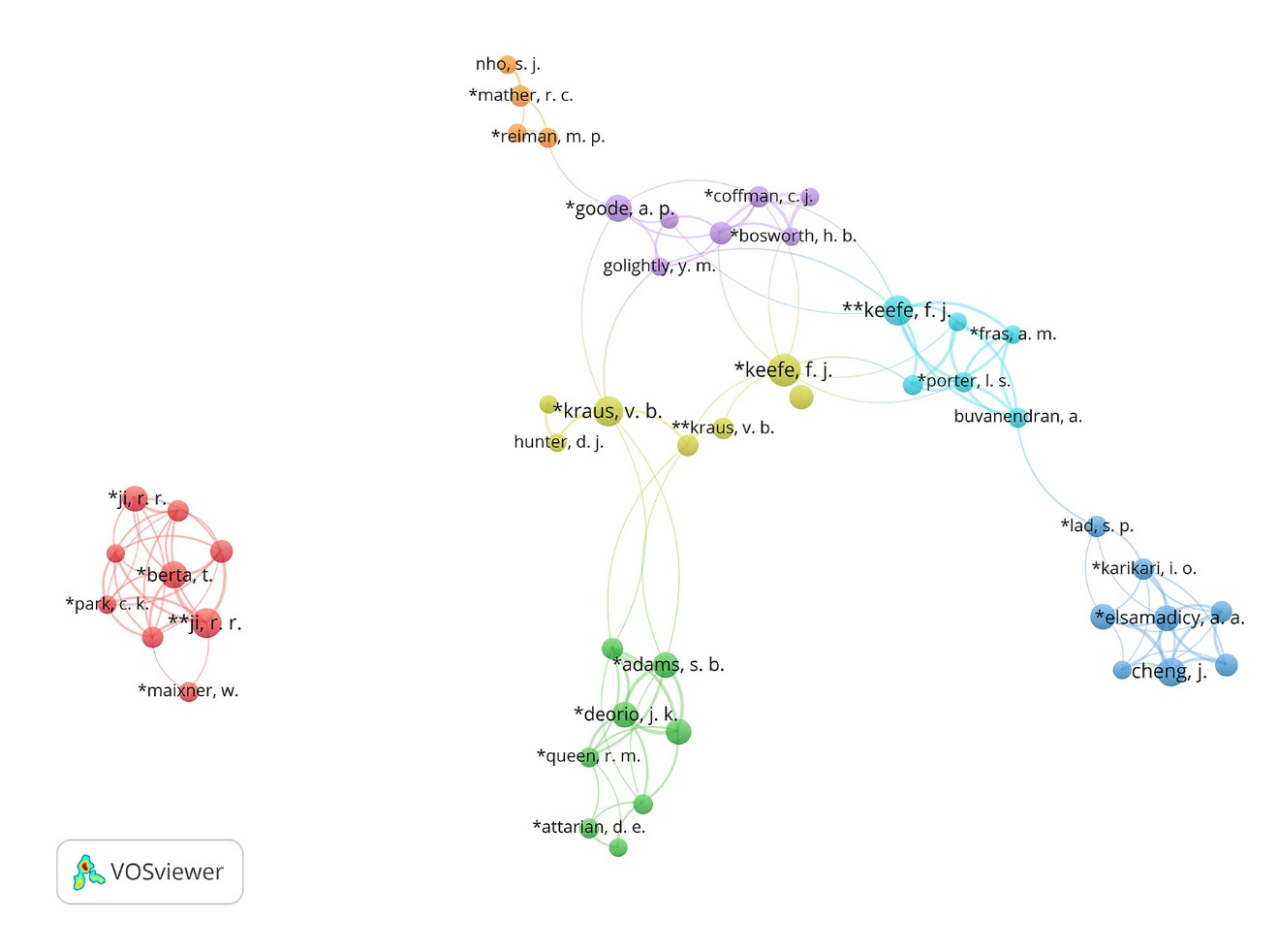
Visualized coauthorship network

Researchers consulted with librarians to identify clusters in the visualizations based on domain knowledge and awareness of Duke author subject specialties (Figure 3). The term map visualization enabled the requesting researchers to identify pain content areas that have been covered by Duke authors and examine interconnectivity through node relatedness. Likewise, the visualized coauthorship network allowed identification of current and potential collaborative partnerships of both Duke and non-Duke researchers. Researchers noted both unconnected author clusters and individual authors acting as bridges between clusters. The VOSviewer visuals and information gleaned from the InCites bibliometric analyses were included in the researchers’ final grant proposal.

**Figure 3:**
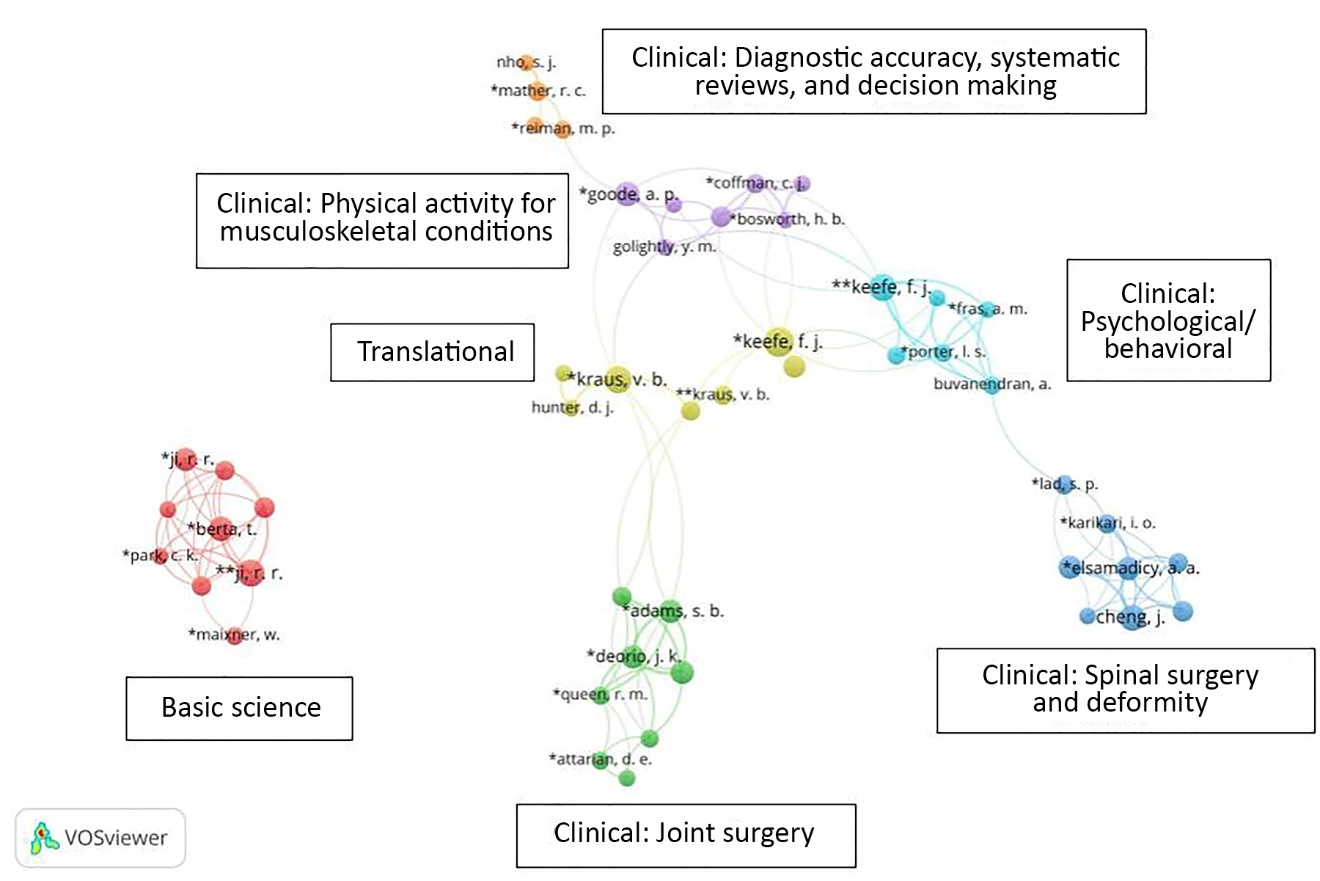
Clusters based on domain knowledge and awareness of Duke author subject specialties

Data visualization and bibliometric mapping are invaluable tools in detecting and analyzing current and potential collaborative research. This project has successfully generated a number of useful visualizations characterizing current and potential collaborations in institutional pain research. The analysis was used in a grant proposal for funding a new pain research center at Duke University and for catalyzing further collaborative research.

The approach described here is an agile option for addressing bibliometric requests where no previous bibliometric service has been established. By pooling skills and resources, librarians were able to address these questions in depth through marriage of preexisting data with novel bibliometric and data visualization tools. At the Duke Medical Center Library, bibliometrics and visualization services have been provided to researchers and research groups in the past via this team-based process on an as-requested basis. These have largely focused on grant groups where publication tracking, research evaluation, and visual story-telling provided support and analysis of current and future research. This service has recently expanded to include development of comprehensive searches for bibliometric analysis of publications generated by specific Duke institutes and divisions and subject area topics to create a visualized state of the art of a particular subject via bibliometric mapping. Both types of requests have continued to increase in volume, and the library continues to develop this service in response to these requests.

**Jordan Wrigley, MA**, jeliwrig@gmail.com, https://orcid.org/0000-0003-0176-5980, Master’s of Science in Library Science Graduate Student, University of North Carolina–Chapel Hill, and Intern, Library & Archives, Duke University Medical Center, 10 Searle Drive, DUMC 3702, Durham, NC 27710

**Virginia Carden, MSLS, AHIP**, virginia.carden@duke.edu, https://orcid.org/0000-0001-9305-6756, Research Impact and EndNote Consultant, Library & Archives, Duke University Medical Center, 10 Searle Drive, DUMC 3702, Durham, NC 27710

**Megan von Isenburg, MSLS, AHIP**, megan.vonisenburg@duke.edu, https://orcid.org/0000-0001-6084-5775, Associate Dean for Library Services & Archives, Library & Archives, Duke University Medical Center, 10 Searle Drive, DUMC 3702, Durham, NC 27710
